# Supplementary material for: Novel Driver Strength Index highlights important cancer genes in TCGA PanCanAtlas patients
Source: PeerJ. 2022 Aug 11;10:e13860. doi: 10.7717/peerj.13860 (PMC9375969; doi:10.7717/peerj.13860)
Supplement: Supplemental Information 2 [file peerj-10-13860-s002.zip › COHORTS/genes plots/cohorts/2021_8_16_14_9_distribution_cohorts_males_HNSC.pdf]

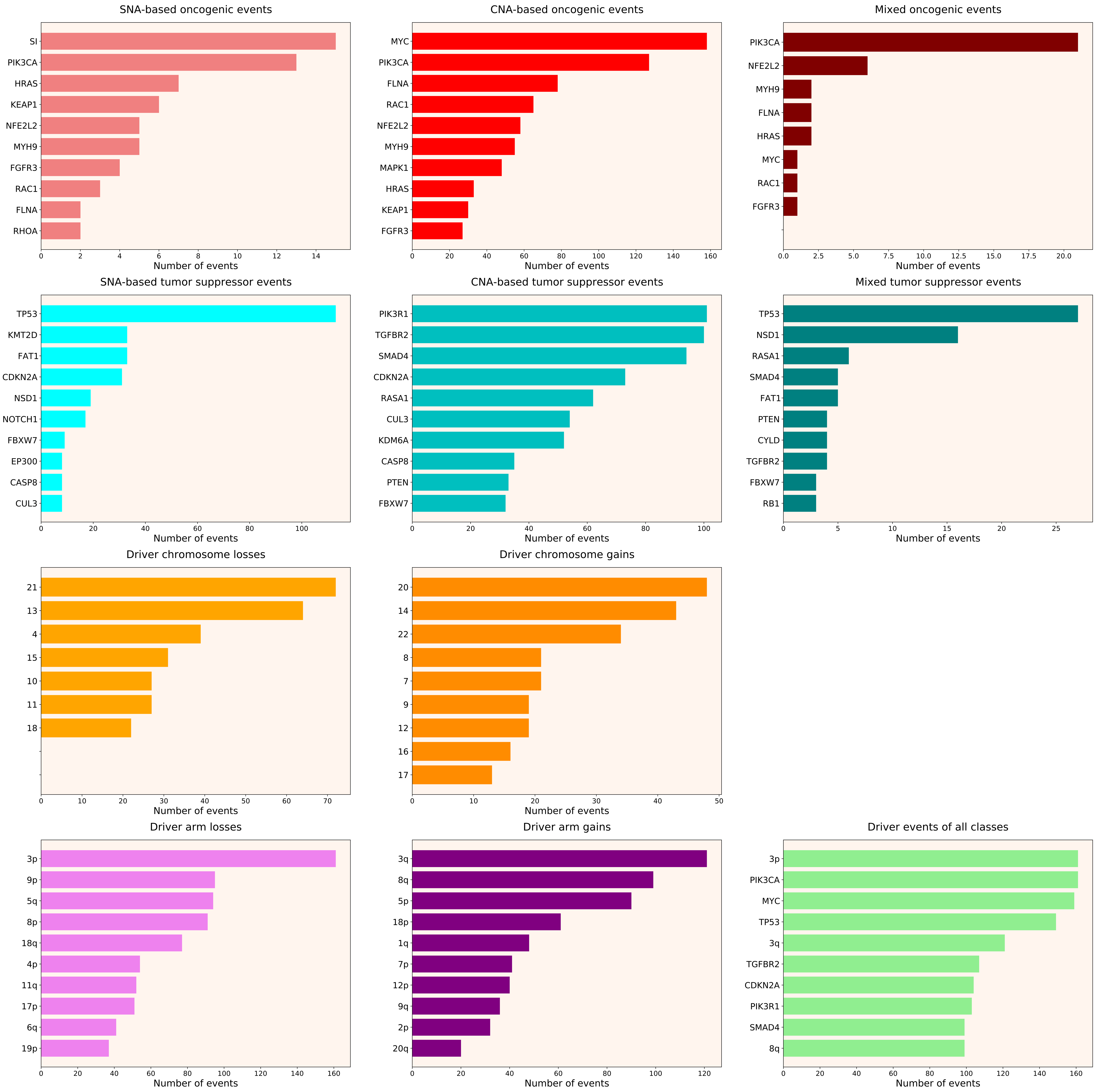

SNA-based oncogenic events
SI
PIK3CA
HRAS
KEAP1
NFE2L2
MYH9
FGFR3
RAC1
FLNA
RHOA
0 2 4 6 8 10 12 14
Number of events
CNA-based oncogenic events
MYC
PIK3CA
FLNA
RAC1
NFE2L2
MYH9
MAPK1
HRAS
KEAP1
FGFR3
0 20 40 60 80 100 120 140 160
Number of events
Mixed oncogenic events
PIK3CA
NFE2L2
MYH9
FLNA
HRAS
MYC
RAC1
FGFR3
0.0 2.5 5.0 7.5 10.0 12.5 15.0 17.5 20.0
Number of events
SNA-based tumor suppressor events
TP53
KMT2D
FAT1
CDKN2A
NSD1
NOTCH1
FBXW7
EP300
CASP8
CUL3
0 20 40 60 80 100
Number of events
CNA-based tumor suppressor events
PIK3R1
TGFBR2
SMAD4
CDKN2A
RASA1
CUL3
KDM6A
CASP8
PTEN
FBXW7
0 20 40 60 80 100
Number of events
Mixed tumor suppressor events
TP53
NSD1
RASA1
SMAD4
FAT1
PTEN
CYLD
TGFBR2
FBXW7
RB1
0 5 10 15 20 25
Number of events
Driver chromosome losses
21
13
4
15
10
11
18
0 10 20 30 40 50 60 70
Number of events
Driver chromosome gains
20
14
22
8
7
9
12
16
17
0 10 20 30 40 50
Number of events
Driver arm losses
3p
9p
5q
8p
18q
4p
11q
17p
6q
19p
0 20 40 60 80 100 120 140 160
Number of events
Driver arm gains
3q
8q
5p
18p
1q
7p
12p
9q
2p
20q
0 20 40 60 80 100 120
Number of events
Driver events of all classes
3p
PIK3CA
MYC
TP53
3q
TGFBR2
CDKN2A
PIK3R1
SMAD4
8q
0 20 40 60 80 100 120 140 160
Number of events
